# Hepatocyte CYP2B6 Can Be Expressed in Cell Culture Systems by Exerting Physiological Levels of Shear: Implications for ADME Testing

**DOI:** 10.1155/2017/1907952

**Published:** 2017-09-10

**Authors:** Timothy G. Hammond, Holly H. Birdsall

**Affiliations:** ^1^Durham VA Medical Center, Research & Development Service, Durham, NC 27705, USA; ^2^Nephrology Division, Department of Internal Medicine, Duke University School of Medicine, Durham, NC 27705, USA; ^3^Space Policy Institute, Elliott School of International Affairs, George Washington University, Washington, DC 20052, USA; ^4^Department of Veterans Affairs Office of Research and Development, Washington, DC 20420, USA; ^5^Departments of Otorhinolaryngology, Immunology, and Psychiatry, Baylor College of Medicine, Houston, TX 77030, USA

## Abstract

Cytochrome 2B6 (CYP2B6) has substantial clinical effects on morbidity and mortality and its effects on drug metabolism should be part of hepatotoxicity screening. Examples of CYP2B6's impacts include its linkage to mortality during cyclophosphamide therapy and its role in determining hepatotoxicity and CNS toxicity during efavirenz therapy for HIV infection. CYP2B6 is key to metabolism of many common drugs from opioids to antidepressants, anesthetics, and anticonvulsants. But CYP2B6 has been extremely difficult to express in cell culture, and as a result, it has been largely deemphasized in preclinical toxicity studies. It has now been shown that CYP2B6 expression can be supported for extended periods of time using suspension culture techniques that exert physiological levels of shear. New understanding of CYP2B6 has identified five clinically significant genetic polymorphisms that have a high incidence in many populations and that convey a substantial dynamic range of activity. We propose that, with the use of culture devices exerting physiological shear levels, CYP2B6 dependent drug testing, including definition of polymorphisms and application of specific inhibitors, should be a standard part of preclinical absorption, distribution, metabolism, and excretion (ADME) testing.

## 1. Introduction

The importance of CYP2B6 in drug metabolism is becoming more and more evident [1, 2]. CYP2B6 metabolizes 2%–10% of clinically used drugs including antineoplastic agents such as cyclophosphamide and ifosfamide, anesthetics such as propofol and ketamine, synthetic opioids such as pethidine and methadone, and antiretrovirals such as nevirapine and efavirenz [1–3]. CYP2B is highly polymorphic [4], but, until recently, difficulties in maintaining its expression in cultured hepatocytes have limited studies on the impact of CYP2B polymorphisms, inhibitors, and inducers, on the dynamic range of its activity [1, 2, 5, 6]. The difficulty in maintaining CYP2B expression also means that the role of this clinically important CYP is largely not addressed in current hepatotoxicity testing. In this review, we will discuss the challenges of culturing hepatocytes, the role of shear stress in cells, and strategies to introduce shear stress into culture systems in order to promote the expression of CY2B.

## 2. Experimental Models Lacking Physiological Stress in Cell Culture Systems

The reliability of current in vitro drug toxicity methods depends on the type of liver cells used and the conditions under which they are cultured [7] (Table 1). Primary human hepatocytes are the FDA agreed-upon gold standard. Several hepatocyte cell lines are available, but all express lower quantities of Phase I and/or Phase II enzymes than do fresh hepatocytes. In a side-by-side comparison, primary human hepatocytes detected 8 of 9 hepatotoxins, whereas the hepatic cell lines HepG2 and HepaRG and the oncogene transfected Upcyte line detected only 6, 3, and 3 of the 9 hepatotoxins, respectively [8]. Generation of hepatocytes from stem cells shows promise [9], but embryonic stem cells have limited availability. Induced pluripotent stem cells from tissues tend to display epigenetic memory with residual expression of genes from their tissue of origin, as well as low expression of Phase I and Phase II enzymes, and a tendency to express fetal genes [7].

Culture conditions have a substantial impact on hepatocyte differentiation and hence the fidelity with which the in vivo models are representative of liver cells in vivo. The current default industry standard is to culture human hepatocytes as a 2D monolayer in tissue culture plates under static conditions [10]. However, when presented with a stiff substrate of tissue culture plastic, whether alone or coated with extracellular proteins such as collagen, hepatocytes dedifferentiate and lose their utility within hours to days [5, 11, 12]. Bell et al. found that 457 proteins, representing 13.9% of the hepatocyte proteome, were significantly altered within the first 24 hours that hepatocytes are plated in 2D monolayers [13].

Sandwiching hepatocytes between layers of gelled extracellular proteins supports cellular polarity, formation of bile canaliculi, and expression of transporter proteins for up to two weeks [7, 14]. However, expression of CYP2C8, CYP2C19, and CYP2D6 decreases with time and, when used as a screening tool, hepatocytes sandwiched in gels have only a 50–60% true positive rate for identification of hepatotoxins [15]. Furthermore, variability between lots of extracellular proteins induces additional variability into the assays [7]. Micropatterned cocultures, in which hepatocytes are surrounded by mouse fibroblasts as feeder cells, have shown better sensitivity at detecting hepatotoxins but have the difficulty of involving cells types from two species [7]. A variety of other organ-on-a-chip platforms show promise for the study of organ-organ interactions but are neither simple, inexpensive, nor scalable for the routine screening of hepatotoxicity [7]. Furthermore, these methodologies support the expression of scant, if any, CYP2B [7].

Hepatocytes in 3D spheroids are an improvement over 2D cultures [7, 13]. Bell et al. found that in comparison to fresh liver cells, there were significant alterations in the expression of 358 proteins in 2D conditions compared to only 132 proteins in 3D spheroids [13]. 3D spheroids can be generated in bioreactors, in hanging drops, or with ultra-low adherence plastic dishes.

Bioreactors stir hepatocytes suspended in media until they aggregate [16]. Bioreactors are ideal for processing of cells in volumes of tens to hundreds of milliliters but are impractical for a screening assay where individual samples need to remain in the range of 100–1000 microliters in order to keep costs low and allow scaling to high throughput systems. Furthermore, bioreactor stirrers generate marked quantities of shear stress and can mechanically damage the hepatocytes that they impact.

Suspension of spheroids in hanging drops solves the problem of low volumes and avoidance of cell fragmentation, but this approach precludes reagent addition and media replenishment during culture, has not been widely validated, and has limited dissemination due to the use of proprietary materials [7].

Primary human hepatocytes self-aggregate into spheroids on ultra-low adhesion cell culture plates [5] and this approach is both economical and scalable. Spheroids generated this manner can be maintained in serum-free media and retain their morphology, viability, and hepatocyte-specific functions for five weeks [5, 13]. Activity of CYP1A2, CYP2D6, and CYP3A4 remains stable over this interval [5]. The sensitivity of hepatocyte spheroids for hepatotoxins increases over the five weeks, rendering them sensitive to toxins in clinically relevant concentrations [13]. 3D hepatocyte spheroids can be cocultured with nonparenchymal cells and can demonstrate liver pathologies such as cholestasis, steatosis, and viral hepatitis [13]. Despite being an improvement in many ways, 3D spheroids generated in this manner still have limitations: larger aggregates have necrotic cores, CYP2C8 activity declines, CYP2C9 increases, and the activity of CYP2B6 has not been determined [5].

## 3. Strategies to Introduce Physiologic Levels Shear Stress in Culture

Each of the culture methods described above misses one key parameter, the ability to expose cells to* physiological* levels of shear stress (Table 1). Hemodynamic flow is well documented to improve rat hepatocyte morphology, function, and metabolic activity in vitro [17–19]. Liver-specific functions, such as albumin synthesis and urea secretion, expression of baseline and inducible Phase I and Phase II enzyme activities, and the capacity to metabolize select drugs, are preserved far longer in 3D bioreactors, which provide shear stress, compared to monolayer systems with no shear stress [19]. Fluid shear also has substantial biochemical and ultrastructural effects on renal cells [20–24]. Shear stress reintroduced into hemodynamic flow systems maintains the expression of certain CYPs [17, 25]. When rat hepatocytes in monolayers were exposed to 0.6 dyne/cm^2^ of shear stress in a perfused Transwell device, CYP1A1 increased 54-fold, CYP1A2 increased 64-fold, CYP2B1 increased 15-fold, and, most importantly for the thesis of this review, CYP2B2 increased threefold relative to static cultures [17]. However, shear must be kept at in vivo levels because effects can be reversed as the applied shear increases [26]. Cultured renal cells also respond to shear stress in and the effects of the shear forces depend greatly on how closely they approximate in vivo levels [24, 27].

Physiological shear can be reintroduced using perfused Transwells [17], hollow fiber culture devices [28], and 3D bioprinting of cells onto perfusable chips [14, 29]. However, these approaches have limited scalability and may require artificial extracellular matrix and growth factors that introduce ill-defined unintended effects. One excellent approach that has yet to be exploited by the pharma industry is the use of suspension culture devices known as rotating wall vessels [30, 31]. The rotating wall vessel is a vertically rotating cylinder of fluid with no headspace and no air. Stress in rotating wall vessels can be delivered at physiological levels by the maintenance of laminar flow conditions [30, 31]. The rotating wall vessel can be spun at a rate to deliver ~0.4 to 0.12 dynes/cm^2^ of shear stress, a value close to in vivo levels [17, 18, 30–33].

## 4. The Role of Shear Stress in Maintaining Cellular Differentiation Including CYP2B

Suspension cultures in rotating wall vessels reinstate many structural and function elements found in vivo [27, 31, 34, 35]. Human renal proximal tubular cells in suspension culture express the endocytic scavenger proteins cubilin and megalin and the associated highly differentiated vesicular transport system not seen in 2D cultures. They also regain the microvilli and tight junctions lost in 2-dimensional culture [34, 35].

**Figure 1:**
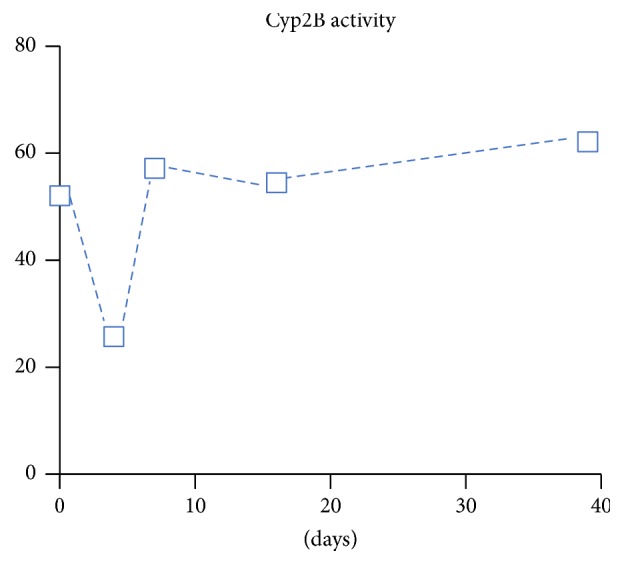
Brown et al. showed that CYP2B1/2, the rat homolog of human CYP2B6, is maintained for at least 39 days in hepatocyte spheroids cultured under physiologic levels of shear stress in rotating wall vessels. CYP2B1/2 activity was measured by generation of pentoxyresorufin-O dealkylase as pmol per total protein in the culture. Adapted from [5] with permission.

Only hepatocyte culture systems with reintroduction of physiological levels of shear have been demonstrated to maintain expression of CYP2B6 [5, 28]. The rotating wall vessel has been shown to maintain hepatocyte CYP2B6 expression for at least 35 days [5]. As shown in Figure 1, hepatocyte spheroids cultured in rotating wall vessels under physiologic shear stress conditions showed an initial dip in CYP2B6 activity in the first week, followed by stable and increasing expression thereafter [5]. Similarly, when primary hepatocytes were perfused to introduce shear in a miniaturized hollow fiber culture device, CYP2B6 levels were partially maintained [28]. In both cases the hepatocytes had well-preserved structural and functional properties, including metabolism of a broad range of drugs.

The maintenance of CYP2B6 activity in hepatocyte cells cultures exposed to physiological shear levels predicates urgent expansion of the scope of testing parameters. New studies should address specific CYP2B6 inhibitors and test bupropion hydroxylation rather than pentoxyresorufin-O-dealkylase activity, as this is the new state of the art for quantifying CYP2B activity.

## 5. Practical Recommendations: CYP2B6 Expression and Polymorphisms in ADME

It is increasingly apparent that many polymorphisms in CYP2B6 are both relatively common and have a substantive effect on rates of drug metabolism [3, 6, 36, 37]. Hence, in screening for CYP2B6-mediated drug metabolism, it is important not only to document expression but also to define the haplotype being studied.

Polymorphisms of CYP2B6 are unusual in several ways. Initial analysis of polymorphisms in the CYP2B6 exon coding region identified nine single base mutations; five are nonsynonymous amino acid changes and four are silent mutations [1, 38]. The number of identified CYP2B6 haplotypes continues to increase over time. The CYP allele homepage (http://www.cypalleles.ki.se/cyp2b6.htm) currently lists 58 haplotypes, 28 alleles, and multiple SNPs with haplotypes to be determined.

The frequency of many CYP2B6 polymorphisms is unusually high [3, 6, 36, 37] but often race-dependent [4, 39]. For example, the C64T, G516T, C777A, A785G, and C1459T mutations were found in 5.3%, 28.6%, 0.5%, 32.6%, and 14.0%, respectively, of 215 Caucasian patients. In a study of 172 Turkish patients, frequencies of three single nucleotide polymorphisms were 28% for G516T, 33% for A785G, and 12% for C1459CT. This is similar to the frequencies found in European populations, but significantly different from that reported for Asian populations [2, 36]. These frequencies are dramatically higher than other CYP450 genetic polymorphisms, where, for instance, CYP2D6 poor metabolizer phenotype is found in 5–10% of Caucasians and only 1-2% of Asians.

Analysis of CYP2B6 polymorphisms should be performed in parallel with analysis of polymorphisms of other CYPs, N-acetyl transferase (NAT1 and NAT2), thiopurine-5-methyltransferase (TPMT), uridine-5 diphosphate glucuronyl transferase (UDP-glucuronyl transferase), Phase II enzymes, and transporters. Several commercial services offer drug metabolism testing on hepatocytes with both wildtype and genetic polymorphisms of selected CYP450 enzymes. This approach needs to be applied to CYP2B6 analysis. Multiplex PCR methods could assay all currently known polymorphisms, but the collapse of sequencing costs allows parallel cost effective analysis of both known and new alleles of all Phase I and Phase II enzymes and associated transporters.

The rotating wall vessel form of suspension culture recapitulates many of the responses needed for drug screening and disease modeling. Initially expensive and limited to large culture volumes during development more than three decades ago, new materials, additive manufacturing, and injection molding manufacturing techniques should allow production of small volume vessels appropriate for high throughput hepatotoxicity testing that are four orders of magnitude cheaper than the original device [27, 31, 34, 35]. Given this dramatic change in feasibility, the rotating wall suspension culture of hepatic cells deserves more thorough exploration and definition so that it can be implemented for drug toxicity screening in high throughput assays.

## 6. Conclusions

CYP2B6 has vast clinical importance. CYP2B6 has frequent polymorphisms that induce a dynamic range of activity, leading directly to hepatotoxicity and mortality during administration of common drugs [40–42]. New suspension culture methods that introduce physiological levels of shear can now maintain expression of CYP2B6 in vitro [5, 33]. Hepatocytes expressing known polymorphisms of CYP2B6 should be a standard part of preclinical metabolism testing. Understanding and assaying cytochrome P450 polymorphisms, especially CYP2B6, will improve drug safety as we evolve to personalized medicine [43].

## Figures and Tables

**Table 1:** Comparison of current in vitro culture systems for hepatotoxicity. Redrawn and edited from Lauschke et al. 2016 [7].

Model	Stability	CYP2B6 expression	Phenotype maintenance	Cell numbers required	Canaliculi formed	Complexity	Comments
2D layer	1-2 days	*Minimal *	Poor	Medium	No	Low	Current gold standard
2D sandwich in gel matrices	2 weeks	*Silent*	Poor	Medium	Yes	Medium	Matrix batches vary
3D spheroids	>5 weeks	*Silent*	Good	Low	Yes	Medium	Cannot add shear stress
Hollow fiber	>5 weeks	*Minimal*	Good	High	Yes	High	Fixed high shear stress
Miniature perfused hollow fiber	>5 weeks	*Present*	Good	Low	Yes	High	Physiological shear stress exerted
Rotating wall vessel	>5 weeks	*Present*	Good	Low	Yes	Medium	Physiological shear stress exerted
